# Identifying driving factors of urban land expansion using Google Earth Engine and machine-learning approaches in Mentougou District, China

**DOI:** 10.1038/s41598-022-20478-z

**Published:** 2022-09-28

**Authors:** Lin-Lin Cheng, Chao Tian, Ting-Ting Yin

**Affiliations:** College of Geoscience and Surveying Engineering, China University of Mining and Technology (Beijing), Beijing, 100083 China

**Keywords:** Urban ecology, Environmental social sciences

## Abstract

The research on driving mechanisms of urban land expansion is hot topic of land science. However, the relative importance of anthropogenic-natural factors and how they affect urban land expansion change are still unclear. Based on the Google Earth Engine platform, this study used the support vector machine classifier to extract land-use datasets of Mentougou district of Beijing, China from 1990 to 2016. Supported by machine-learning approaches, multiple linear regression (MLR) and random forests (RF) were applied and compared to identify the influential factors and their relative importance on urban land expansion. The results show: There was a continuous growth in urban land expansion from 1990 to 2016, the increased area reached 6097.42 ha with an average annual rate of 8.01% and average annual intensity rate of 2.57%, respectively. Factors such as elevation, risk of goaf collapse, accessibility, local fiscal expenditure, industrial restructuring, per capita income in rural area, GDP were important drivers of urban land expansion change. The model comparison indicated that RF had greater ability than MLR to identify the non-linear relationships between urban land expansion and explanatory variables. The influencing factors of urban land expansion should be comprehensively considered to regulate new land policy actions in Mentougou.

## Introduction

Land use/cover change (LUCC) has become the focus of land science research^1^. As an important component of LUCC, urban land expansion plays a vital role in the regional ecological environment and economic development^2^. As the largest developing country in the world, China has experienced rapid urban land expansion since the implementation of reform and opening-up in 1978. However, the loss of natural vegetation^3^, farmland^4^, as well as environmental pollution^5^ and other problems are increasingly prominent in response to urban sprawl^6^. Thus, in order to reduce the negative impact of urban land expansion on the social environment, there is a great need to clarify the driving mechanisms of urban land expansion^7^.

In the past decades, scholars have intensively investigated urban land expansion, which is mainly focused on the following aspects: (i) the characteristics of urban land expansion and spatial distribution trend of urban land expansion and (ii) analysis drivers of urban land expansion^8,9^. In the first research area, previous studies generally used urban land expansion density^2^, urban land expansion intensity^10^, gravity center migration^11^ and Gini coefficient^7^ to evaluate urban land expansion change.

For the second research focus, urban land change is a complicated process that involves the spatial and temporal of various natural and socio-economic factors, therefore, the driving factors often have strong regional and spatial heterogeneity. Wu et al.^12^ found that urban land expansion was closely related to transportation accessibility. Thus, distance to roads, distance to city center and distance to rivers had been used as variables in some studies of urban land expansion^13,14^. Slavati^15^ indicated that the effect of neighborhood and increased population density drove the urban land expansion in many Chinese cities. Other factors such as topography and geological conditions were found to be meaningful drivers of urban land expansion^6,16^, with flatter, lower elevation and shadier areas being more prone to development as construction land^17^, especially in areas where mineral resources have been exploited for a long time, the geological environment is poor, such as goaf collapse area and surface subsidence^18^. The land unit is probably to convert into urban land if it is surrounded by more urban land, thus, neighborhood factors are considered as the main factors affecting urban land expansion in many studies^19,20^. Land market factors have also been linked to urban land expansion in many areas. Specifically, construction land utilization efficiency^21^, per capita GDP^19^ and population urbanization rate^22^ have positive effects on urban land expansion, such as urban redevelopment and road network expansion^23^. Additionally, with the increased proportion of urban population, managers and citizens are accelerating the pace of urbanization^24^. Relevant government drivers, such as industrial restructuring and local fiscal expenditure^23^, can greatly affect urban land expansion. Methodologically, the structure equation models^16,24^, regression analyses^25,26^ and statistical analyses^6^ were generally used to explore the relationship between urban land expansion and its drivers, and further investigated the driving mechanism of urban land expansion. Although extensive research on urban land expansion analyses, the complexity of relative importance of influential factors and how they affect urban land expansion are still unclear. Traditional statistical models of urban land also have their limitations. These models generally utilize correlation and regression coefficients to assess variable importance. The results may be affected by the cross-correlated variables, which may lead to inadequate and misleading results^27^. Further, the monitoring of land cover datasets is traditionally obtained through field measurement^28^, geographic information system^19^ and remote sensing^29^, the pre-processing of these datasets is complex, making it difficult to continuously monitor and update land-use data for a long time. Therefore, it is necessary to improve the methods of traditional long-term land use datasets processing in some effective ways.

To solve these issues mentioned above, the study investigated the relative importance by considering the relationships between natural and human factors for urban land expansion. More specifically, the objective of this study is to reveal the dynamic evolution of urban land expansion in Mentougou over the past 26 years, as well as the basic processes and identify the ranking of the main driving factors of urban land expansion. For this purpose, supported by the Google Earth Engine (GEE) platform and machine-learning approach, our study selected the long-term series remote sensing images, and used Support Vector Machine (SVM) classifier to extract land use datasets, both multiple linear regression (MLR) and random forest (RF) models were used to evaluate the potential contribution of topographical, geological, accessibility, neighborhood, land market and government action factors to urban land expansion in Mentougou district of Beijing, China. Each approach was applied to the land use data and the results of the two models were analyzed and compared. Furthermore, the driving factors and their importance to urban land expansion were revealed.

## Materials and methods

### Study area

**Figure 1:**
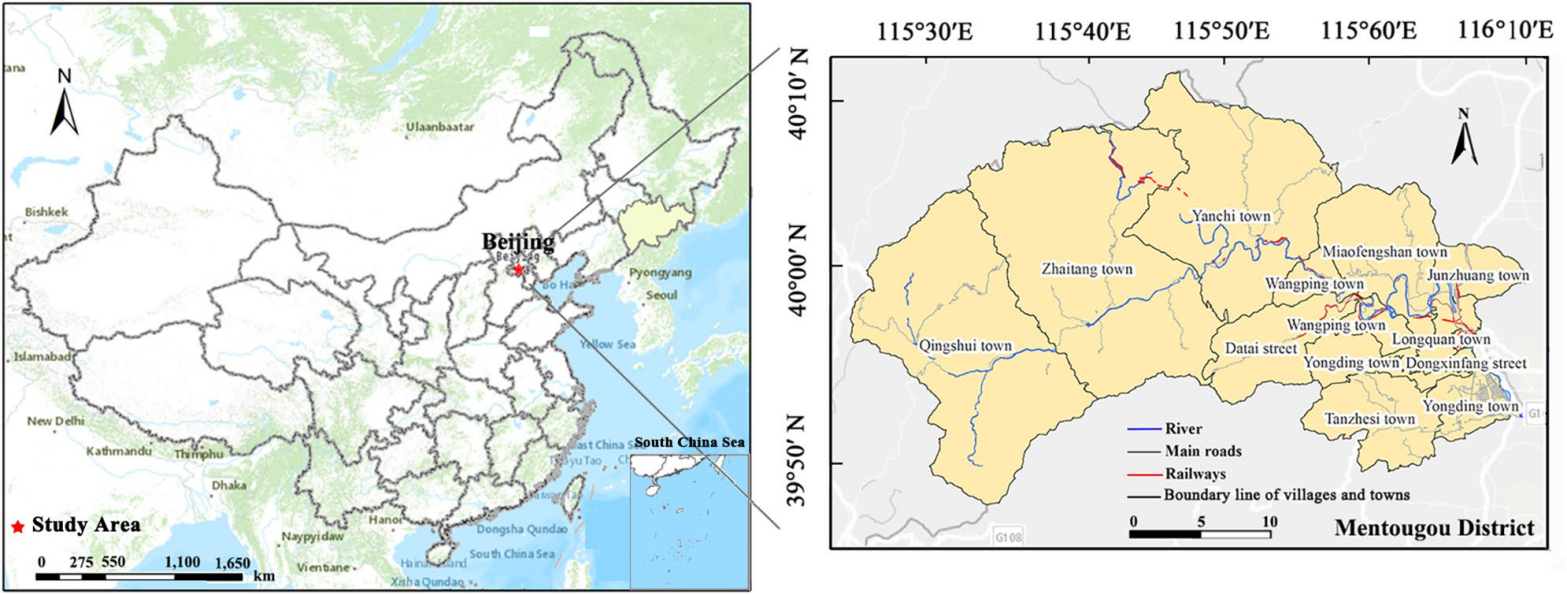
Location of the study area (the map was created by the author using the software ArcGIS 10.5, www.esri.com).

Mentougou district is located in the west of Beijing City, China (39°48′–40°10′ N and 115°25′–116°10′E) and covers an area of 144 892 km^2^, of which mountainous area constitutes about 98.5% (Fig. 1). It is characterized by complex surface geological conditions, undulate topography, and is rich in vegetation resources and belongs to the deciduous broad-leaved forest type area. Historically, there were a lot of mineral resources, which were the main economic income of local industries. However, as it was recognized as Beijing's Ecological Conservation Area in 2004^30^, Mentougou faced industrial transformation^31^, the local land use experienced a significant change^18^. With the acceleration of the process of urbanization, the area has undergone intense urban land expansion, with GDP and population growing from 7.51 billion yuan (RMB) and 0.25 million in 2000 to 17.45 billion yuan (RMB) and 0.32 million in 2016. In addition, many abandoned mined land has been reclaimed and utilized as the closure of various mines, which also provided the area with the impetus for rapid urban expansion^32^.

### Data resources

#### Urban land area

Urban land area refers to the developed land covered by impervious surfaces, including urban–rural land, residential land, industrial and mining land, transportation land^7^. In this study, we classified land use types into arable land, forestland, grassland, urban land and unused land. Google Earth Engine (GEE) platform provides the possibility for large-scale remote sensing data processing and mining analysis^33^. Therefore, Landsat TM and ETM images from 1990 to 2016 were selected as data sources and archived in the GEE. These images were obtained on April 19 and May 19, 1990 obtained from Landsat TM, on April 19, 2000 and April 21, 2010 from ETM and TM, and April 30 and May 19, 2016 from Landsat TM. The spatial resolution of the land use dataset was 30 m and the temporal resolution was per 16 days. All images were obtained from plant growing seasons, in order to facilitate comparative analysis. We also obtained land use datasets from the Data Center for Resources and Environmental Sciences, Chinese Academy of Sciences (RESDC) (http://www.resdc.cn), which has high accuracy and application value^32^.

#### Explanatory factors: selection and pre-processing of variables

Based on the data available and literature review in the introduction section, driving factors (independent variables) that are likely to affect urban land expansion were classified into 6 categories, including topographical, geological, accessibility, neighborhood, land market and government action factors, and urban land expansion change density was selected as dependent variables^14,31^.

(i) *Topographic factors* High resolution (30 m) Digital Elevation Model (DEM) data, developed in the 1980s, collected from the National Administration of Surveying, Mapping and Geoinformation of China. Based on DEM, the terrain factors were extracted to obtain the corresponding slope and aspect. Among them, the aspect factor was further converted into aspect index^27^, the formula is as follows:
  $${\text{Aspect index}} = {\text{cos}}(q \times \pi /{18}0)$$
  where *θ* represents the aspect (value range is 0°–360°), and the aspect index ranges from − 1 to 1. The potential solar radiation is higher when the index value is closer to 1.
(ii) *Geological factors* Geological factors include the risk of goaf collapse and fault. The data of the year 2010 were available from our project of *Optimization Technology for the Reuse of Abandoned Mined Land in Industrial Succession Cities*^18^. The data generating process was also based on the results of our research.
(iii) *Accessibility factors* Four accessibility variables such as distance to main roads, distance to railways, distance to city center and distance to rivers were extracted from National Administration of Surveying, Mapping and Geoinformation of China (http://218.244.250.78/NgccDigitalHall/). The data in 2000, 2010 and 2016 were used for main roads and railways. Other factors present no significant dynamic throughout the study period, thus, data from the year 2000 was used to calculate other accessibility factors.
(iv) *Neighborhood factors* Based on the previous study^7^, the proportion of urban land area within a 7 × 7 object pixel was set for each pixel in the study area. The neighborhood factor is calculated by Block Statistics Tool in ArcGIS. We calculated the neighborhood factor in 1990, 2000, 2010 and 2016, respectively.
(v) *Land market factors* Land market factors include utilization efficiency of construction land, population urbanization rate, per capita GDP and per capita rural income. The proportion of non-agricultural output value to the area of construction land and urban resident population were used to calculate construction land utilization efficiency and population urbanization rate, respectively. The data were collected from the Statistical Year Book of Beijing City (http://tjj.beijing.gov.cn/) from 1990 to 2016.
(vi) *Government action factors* Government action factors include industrial restructuring and local fiscal expenditure. The industrial restructuring was represented by the proportion of the tertiary industry output value to the secondary industry output value. The government action factors were also obtained from the Statistical Year Book of Beijing City from 1990 to 2016. Details of these variables are provided in Table 1.

**Table 1:** Description of driving factors.

Variable type	Variable name	Minimum/maximum	Unite	Code	Resolution/scale
Topographic factors	Elevation	51/2274	m	Elev	Raster/30 m
	Slope	0/68.45	°	Slope	Raster/30 m
	Aspect	− 1/1	%	Aspect	Raster/30 m
Geological factors	Fault risk	0/1	–	Fault	Raster/30 m
	Risk of goaf collapse	0/1	–	Colla	Raster/30 m
Accessibility factors	Distance to main roads	0.15/11.25	km	Dis_road	Vector/1:50 000
	Distance to railways	0.02/32.55	km	Dis_railway	Vector/1:50 000
	Distance to city center	0.01/51.38	km	Dis_city	Vector/1:50 000
	Distance to rivers	1.48/12.70	km	Dis_river	Vector/1:50 000
Neighborhood factors	Neighborhood function	0/1	–	Neig_function	Vector/1:50 000
Land market factors	Utilization efficiency of construction land	0/45.53	Million RMB/ha	UCL	Vector/1:50 000
	Population urbanization rate	0/55.25	%	PUR	Vector/1:50 000
	Per capita GDP	279/65,620	RMB/ha	CGDP	Vector/1:50 000
	per capita rural income	6005.73/31,470.4	Person/ha	Rural income	Vector/1:50 000
Government action factors	Industrial restructuring	0.77/1.18	–	IndRest	Vector/1:50 000
	local fiscal expenditure	9.38/29.54	Million RMB	Fis_expenditure	Vector/1:50 000

### Research procedure

**Figure 2:**
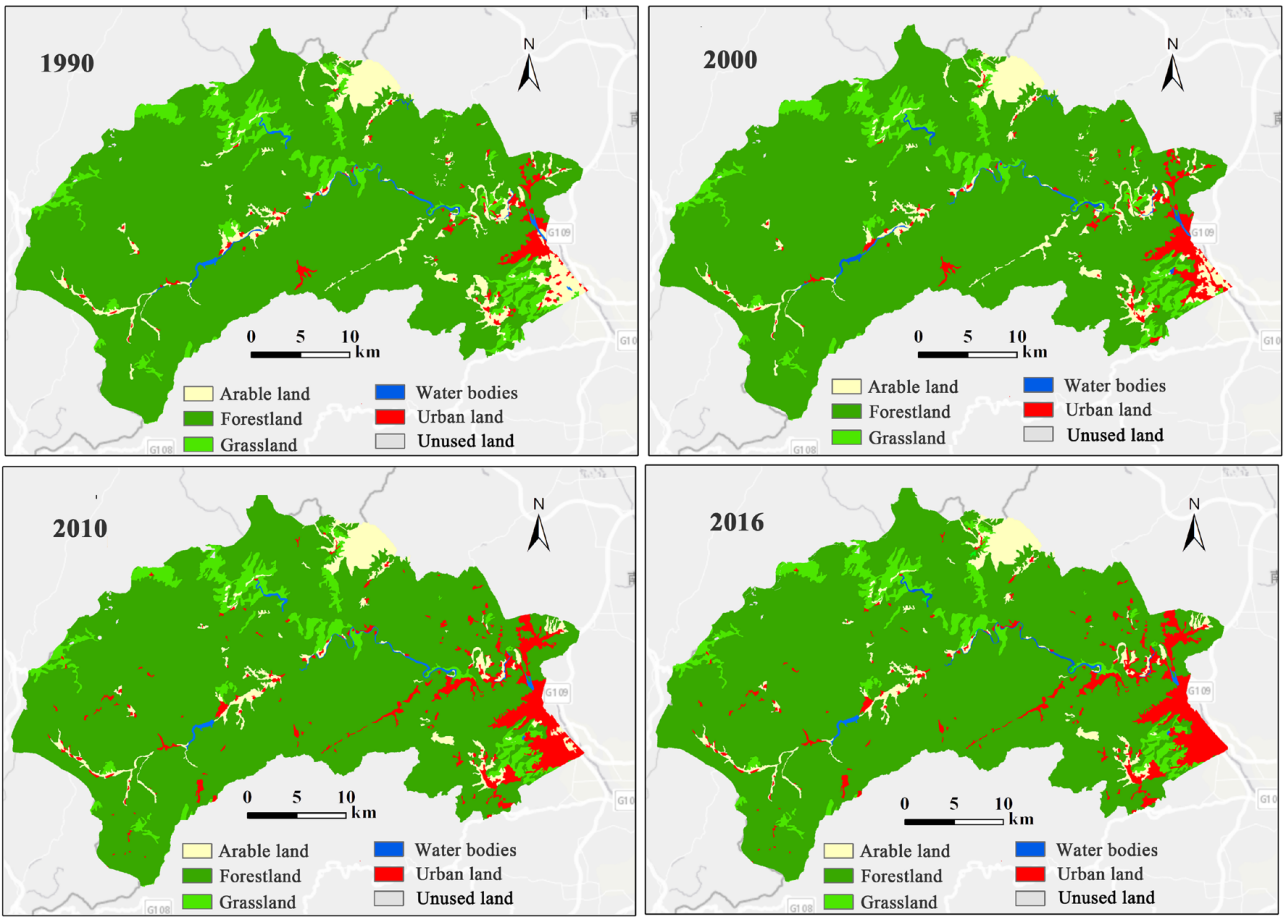
Land cover maps in Mentougou from 1990 to 2016.
(iv) Each explanatory variable was generated as a layer in the ArcGIS environment and converted to a 30 × 30 m grid for model-fitting. In order to reduce the error of samples distribution on the results, the expansion intensity of urban land was taken as the dependent variable. the original driving factors data were randomly divided into two parts: training samples (60%) and validation samples (40%)^36^. All samples were divided repeatedly for 5 times to obtain stable results, resulting in 5 random sub-samples of the data. MLR and RF models were then applied and compared to identify the influential factors and their relative importance on urban land expansion, the partialPlot of RF model was used to calculate the influence intensity (variable effect) on urban land expansion change. The research framework was shown in Fig. 3.

(i) Supported by the GEE platform, we firstly selected Landsat TM and ETM images with cloud-free images from 1990 to 2016, and extracted features from the land use datasets. These pre-processed images were cropped according to the study area boundaries to obtain the land use datasets for 1990, 2000, 2010 and 2016, and then LULC change covering the whole region was assessed.
(ii) Referred to the interpretation principles^34^, we performed a visual analysis of land use dataset classified pixels, and randomly selected an average of 10 000 pixels (about 6000 (60%) pixels for training samples and 4000 (40%) for verification samples) for sample analysis from each year, which was used for evaluating the classified maps. For more information about data processing, see Appendix Table 1.
(iii) The LULC results were extracted by the SVM classifier. The area of different land use types was shown in Appendix Table 2. The indicators of overall accuracy and kappa coefficient were calculated from the error matrix^35^ to evaluate the overall classification accuracy, and land use data of RESDC was used as standard. The overall accuracy and Kappa coefficient of LULC classification from 1990 to 2016 were 0.84 ± 0.03 and 0.83 ± 0.06, respectively (Fig. 2). The overall accuracy of each land use type was above 0.82 ± 0.05, and the Kappa coefficient ranged from 0.84 ± 0.06 to 0.87 ± 0.07 (Table 2). The results indicated that the overall accuracy of the SVM classification is high, which meets the research needs. The area of different land use types refers to Appendix Table 2.

**Table 2:** The accuracy assessment of land use interpretation from 1990 to 2016.

Type	Arable land	Forestland	Grassland	Water bodies	Urban land	Other land	Overall
Accuracy	0.82 ± 0.05	0.86 ± 0.03	0.85 ± 0.03	0.84 ± 0.02	0.87 ± 0.09	0.86 ± 0.04	0.84 ± 0.03
Kappa coefficient	0.84 ± 0.06	0.87 ± 0.04	0.86 ± 0.04	0.85 ± 0.03	0.86 ± 0.08	0.87 ± 0.07	0.83 ± 0.06

**Figure 3:**
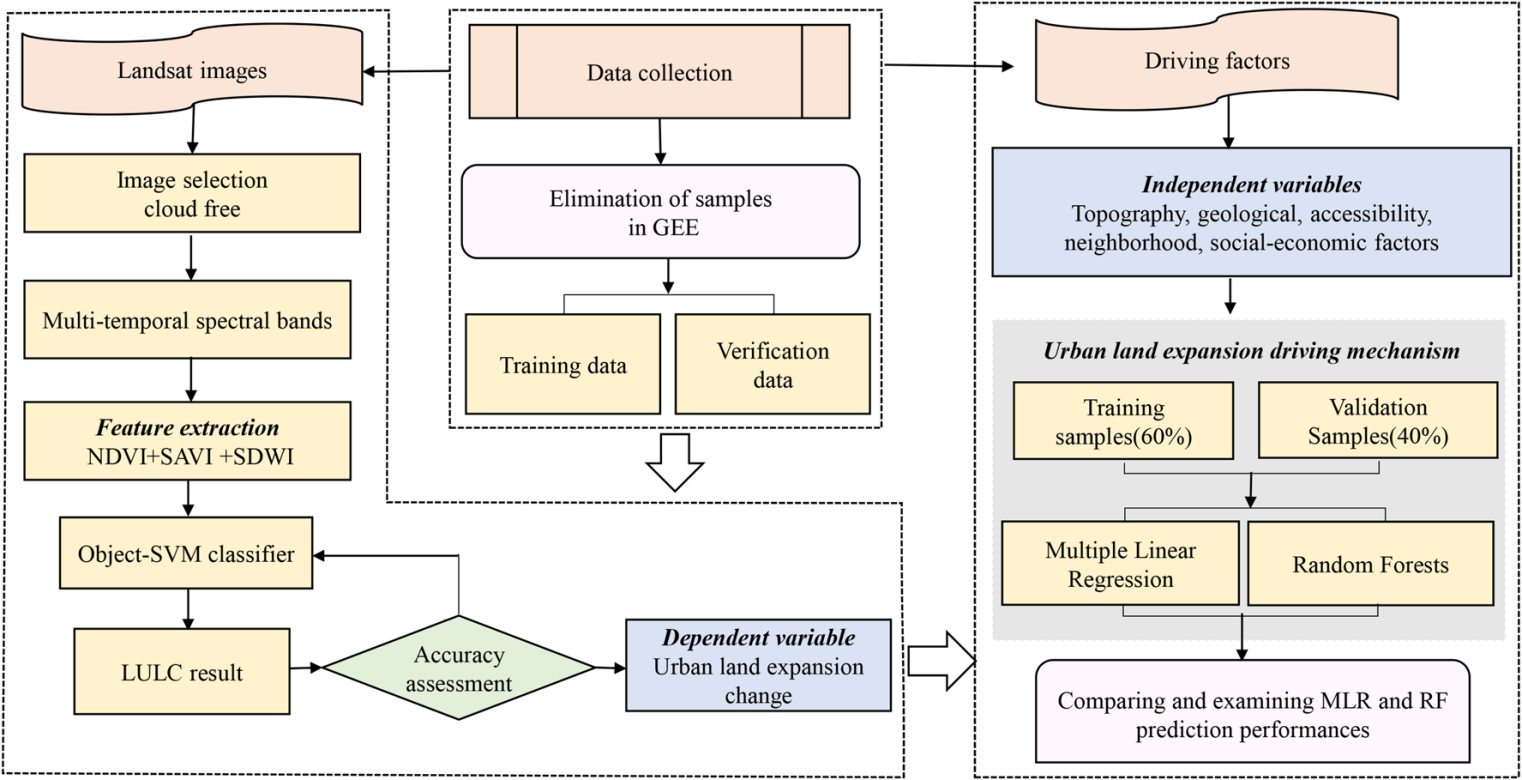
Schematic representation of the research framework. SAVI, NDVI, NDW and LULC represent Soil Adjusted Vegetation Index, Normalized Difference Vegetation Index and Normalized Difference Water Index and land use/cover.

### Models and computing procedures

#### Urban land expansion

The average annual urban expansion rate (UE) and average annual urban expansion intensity rate (UI) are used to compare urban land expansion in different periods^6^. The equation of average *UE* is as follows:

2 $$UE = \frac{{U_{b} - U_{a} }}{{U_{a} }} \times \frac{1}{T} \times 100\%$$

The average *UI* is expressed as follows:

3 $$UI = \frac{{U_{b} - U_{a} }}{{U_{b} }} \times \frac{1}{T} \times 100\%$$

where *U*_*a*_ and *U*_*b*_ represent the total urban land area in the initial and final time period, respectively, and T is the time period.

#### SVM-based classification

There are many automatic land classification algorithms, such as statistical analysis method, decision tree method, K-means algorithm, random forest regression (RF), Super Vector Machine (SVM)^37^. Among these algorithms, SVM is a machine learning method based on non-probabilistic binary functions^38^. Compared to most classification methods, SVM is characteristic of high fitting ability, high predictive power and high-dimensional feature space, which has been widely used in remote sensing image classification research in recent years^37,39^.

SVM-based classification aims to find a low-dimensional classification hyperplane in the constructed high-dimensional space based on a kernel function, which is typically adopted polynomial basis, Gaussian radial basis and sigmoid basis functions^37^. Among these functions, previous studies have proved that the gaussian radial basis works best in most situations^40,41^. Therefore, we chose the Gaussian radial basis function as the SVM classifier, and selected the two necessary parameters of the kernel width γ and the penalty parameter C, which were directly affected the classification accuracy. Based on relevant studies, the result will be better when C takes 100 and γ takes the reciprocal of the number of bands^42^. In this study, the number of Landsat TM/ ETM bands involved in the classification of images was 9, so the kernel width was set 1/9 and the penalty parameter was set 100.

### Models

#### Multiple linear regression

Multiple Linear Regression (MLR) has been widely applied in the study of driving factors of land use change^23,43^. In order to eliminate the factors with collinearity, the Variance Inflation Factor (VIF) was used to test the influencing factors before fitting MLR model^44^. We randomly selected training samples and validation samples for 5 times of calculations, and the predictive ability of the model was tested based on the validation data. The statistically significant predictor variables (α = 0.05) were chosen from at least three of five training samples to fit the final MLR.

The LMG metric (Lindeman, Merenda, and Gold method), which identifies the contribution of variables and other predictors^32^, and represents the average R^2^ contribution of each variable in the regression. In our study, the relative importance of each variable was measured and evaluated by LMG index. The higher value of LMG metric shows the higher importance of the variables, and variables less than 2% LMG were removed from the final model^45^. The LMG metrics were calculated in the Relaimpo package of R software.

#### Random forest model

Random Forests (RF) is a non-parametric algorithm, which can explore the relationship between independent variables and dependent variables, and calculate the relative importance of covariates^46^. In this study, the bootstrap resampling technique was used to randomly extract ‘ntree’ sampling with sample size of N from urban land expansion data^27^, so as to randomly select the number of variables used at each split of ‘mtry’ variables on node of each classification tree. The RF is composed of ‘ntree’ trees, and the results are the average of all ‘ntrees’. In each sampling, the results accounted for about two-thirds of the entire sample, and retains the rest of samples (called out-of-bag, OOB) to estimate the internal error^47^ (Pang et al., 2020). We set the ‘mtry’ as 4 and ‘ntree’ as 2000 in the number of independent variables. Finally, variable importance was extracted based on node impurity, which was calculated based on the decline in the Mean Decrease Accuracy (%IncMSE) of prediction after splitting nodes.

%IncMSE in RF method was used to measure the importance of independent variables in urban land expansion. According to the average calculation results of 5 samples, we selected the most relevant variables to fit the final model. Besides, the Partial Plot Function in R statistical software package was used to build part of the correlation diagram and analyze the main driving factors of urban land expansion. And then the coefficient of determination (R^2^) and mean absolute error (MAE) were used to test the fitting accuracy of MLR and RF^48^. A higher value of R^2^ and a lower value of MAE indicated a higher model interpretation accuracy. The driving factors analyses of RF were carried out by R statistics software.

## Results

### Urban land expansion dynamic change

**Figure 4:**
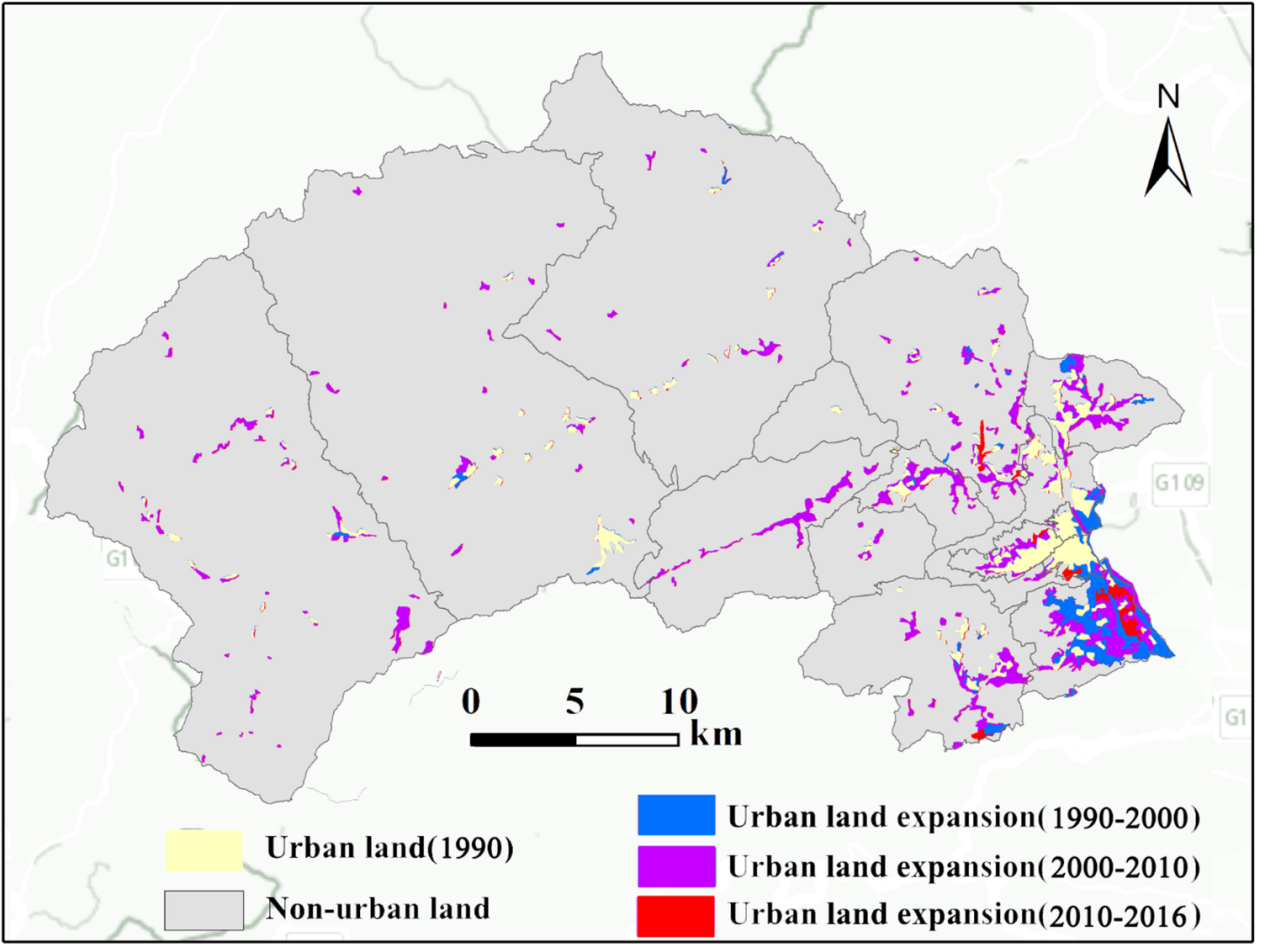
Spatial expansion distribution of urban land from 1990 to 2016.

The overall urban land expansion in Mentougou from 1990 to 2016 is shown in Fig. 4. Urban land was mainly distributed in shallow mountains and hilly areas. In 1990–2000, the urban land expanded with an average *UE* of 3.89% and average *UI* of 2.80% and the increased area of 1127.19 ha, which were concentrated in the southeast of Mentougou, the main urban land area. In 2000–2010, the urban land expansion rate and intensity were the highest with an average *UE* of 10.11% and average of *UI* 5.02% and the increased area of 4072.64 ha, the urban core expansion became more apparent, urban expansion was mainly along the main transportation lines. From 2010 to 2016, under the background of industrial transformation and urban planning, the average *UE* and average *UI* increased slowly, with 1.85% and 1.66%, respectively. The total urban land expansion from 1990 to 2016 showed a continuous upward trend and increased 6097.42 ha with an average *UE* of 8.01% and average *UI* of 2.57%*,* respectively (Table 3).

**Table 3:** Expansion of urban land, average expansion rate (UE), an average expansion in-tensity (UI), 1990–2016.

Period	Expansion of urban land (ha)	Average *UE*(%)	Average *UI*(%)
1990–2000	1127.19	3.89	2.80
2000–2010	4072.64	10.11	5.02
2010–2016	897.59	1.85	1.66
1990–2016	6097.42	8.01	2.57

### Variable importance measurement

#### Multiple linear regression

The results of the multicollinearity test (Table 4) showed that no obvious collinearity was found among all the variables, and 16 variables were used as influencing factors for MLR model fitting. It can be seen from Table 5 that local fiscal expenditure, industrial restructuring, per capita rural income and per capita GDP were positively correlated with the urban land expansion in Mentougou. The importance of the independent variables showed that the most important factors in the MLR model were the elevation, risk of goaf collapse, then followed by distance to city center, local fiscal expenditure, industrial restructuring, distance to main roads, per capita rural income. While population urbanization rate and per capita GDP had a lower impact on urban land by the MLR model. The final model was built with 9 variables in the dataset.

**Table 4:** Significance of independent variables of 5 intermediate models and the importance of variables based on LMG (%) in MLR. “No. sample sig.” represents the number of samples where each variable is significant.

Variable	*p* Value Min	*p* Value Max	No. Sample Sig	VIF	LMG(%)
Elev	< 0.001	< 0.001	5	6.531	22.355
Collapse	< 0.001	< 0.001	5	6.914	18.995
Dis_city	< 0.001	0.032	5	2.042	11.442
Fis_expenditure	< 0.001	< 0.001	5	2.096	11.392
IndRest	< 0.001	< 0.001	5	1.733	11.039
Dis_road	< 0.001	< 0.001	5	1.778	10.747
Neigh	0.476	0.822	0	4.321	8.049
Dis_railway	0.095	0.462	0	4.159	5.283
Dis_river	< 0.001	< 0.001	5	1.545	3.31
Rural income	< 0.001	< 0.001	5	1.174	2.602
UCL	< 0.001	0.424	0	5.734	2.306
PUR	< 0.001	0.003	5	3.331	2.301
CGDP	< 0.001	0.043	5	5.698	2.205
Slope	0.009	0.409	0	1.145	1.101
Fault	0.091	0.464	0	1.345	0.846
Aspect	< 0.001	0.791	0	1.261	0.194

**Table 5:** Results of the MLR model with 9 selected variables.

Variables	Estimate	Std.Error	T-test	*p* value	LMG(%)
Intercept	0.400	0.122	3.283	< 0.001	
Elev	< 0.001	< 0.001	− 9.982	< 0.001	19.69
Collapse	− 0.01	< 0.001	− 7.691	< 0.001	15.35
Dis_city	< 0.001	0.029	− 10.659	< 0.001	13.86
Fis_expenditure	0.2	0.057	3.53	< 0.001	13.79
IndRest	− 0.01	< 0.001	4.011	< 0.001	13.61
Dis_road	− 0.01	< 0.001	− 6.812	< 0.001	13.18
Rural income	0.014	0.003	4.758	< 0.001	5.98
PUR	− 0.014	0.003	− 4.298	< 0.001	5.87
CGDP	− 0.001	< 0.001	4.225	< 0.001	4.46

#### Random forest regression

**Figure 5:**
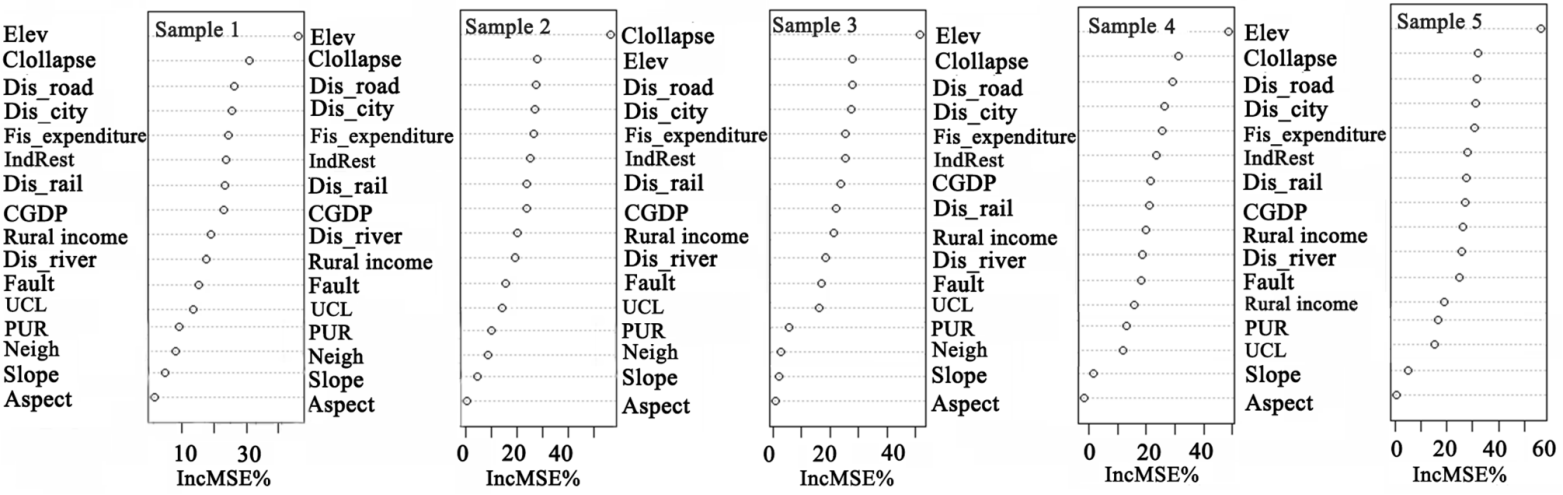
Importance ranking of driving factors by RF model.

According to the research results, we took 20% as the threshold of the %IncMSE, and selected the significant variables with %IncMSE higher than 20% for the final model. Figure 5 is the ranking of the impact of each variable on urban land expansion. The final model was built with the 9 variables shown in Table 6. In this model, the %IncMSE value in descending order was as follows: elevation, risk of goaf collapse, distance to main roads, distance to city center, local fiscal expenditure, industrial restructuring, distance to railways, per capita GDP, per capita rural income.

**Table 6:** The RF model with selected, presented variable importance in descending order based on % IncMSE.

Variables	Variable importance (%IncMSE)
Elev	49.98
Collapse	29.45
Dis_road	27.73
Dis_city	27.21
Fis_expenditure	25.78
IndRest	25.72
Dis_rail	24.51
CGDP	22.03
Rural income	20.92

### Model fitting comparison

The fitting results of both MLR and RF showed that all variables appeared as highly significant (*p* < 0.001) in the intermediate models. Table 7 showed the adjusted R^2^ of each training sample and the correlation between observed and predicted values in MLR. Among them, the adjusted R^2^ values were 0.57–0.59, the correlation values were all above 0.763, and the final model interpretation variance percentages were above 58%. In contrast, RF modelling showed a proportion of explained variance between 88.73% and 89.34% in both training and validation datasets. The mean square residual error was 0.00861–0.00868, and the correlation between the observed value and the predicted value was higher than 0.928 (Table 8).

**Table 7:** Adjusted R^2^ and correlation between observed (Obs) and predicted (Pre) values in MLR.

Sample	Adjusted R^2^	Correlation Obs vs Pre	Significance	Variance explained (%)	
				Training data (60%)	Validation data (40%)
sample1	0.59	0.765	*p* < 0.001	58.41	59.21
sample2	0.57	0.763	*p* < 0.001	58.12	58.27
sample3	0.58	0.764	*p* < 0.001	58.33	58.23
sample4	0.57	0.763	*p* < 0.001	58.25	58.16
sample5	0.57	0.763	*p* < 0.001	58.34	58.13

**Table 8:** Results of RF model and correlation between observed and predicted values.

Sample	Residual R^2^	Correlation Obs vs. Pre	Significance	Variance explained (%)	
				Training data (60%)	Validation data (40%)
sample1	0.00869	0.929	*p* < 0.001	88.84	89.12
sample2	0.00861	0.931	*p* < 0.001	88.24	89.34
sample3	0.00862	0.928	*p* < 0.001	88.83	88.78
sample4	0.00862	0.935	*p* < 0.001	88.73	88.90
sample5	0.00865	0.934	*p* < 0.001	88.85	88.91

**Figure 6:**
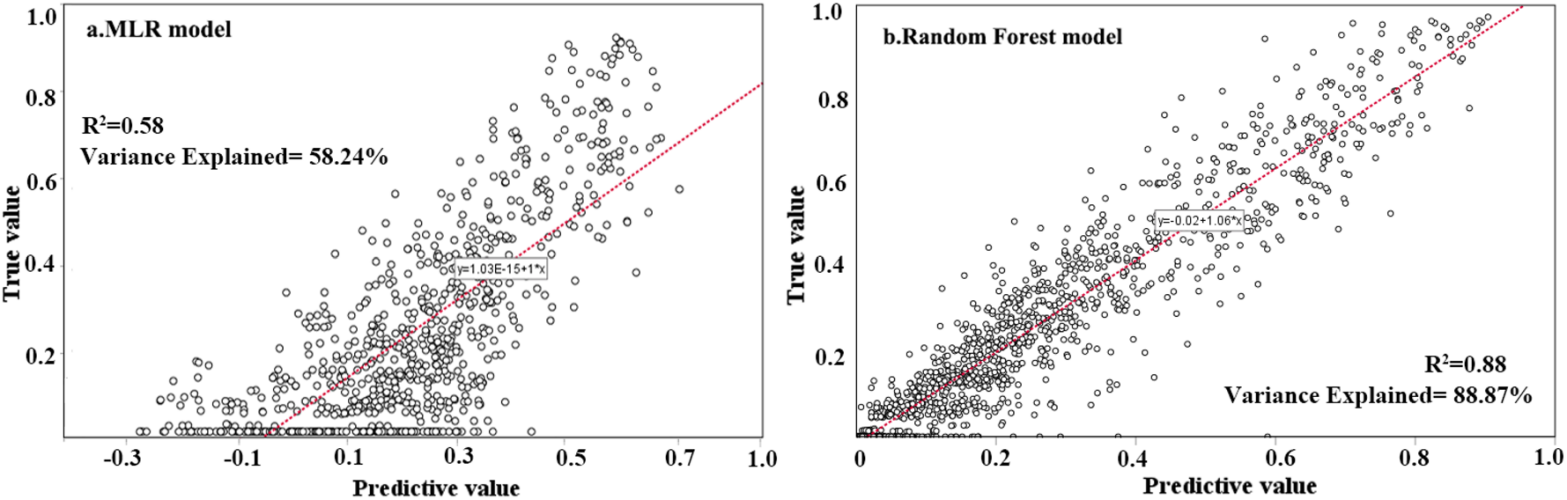
Observed and predicted values by MLR and RF Models.

Figure 6 showed R^2^ and model interpretation rate of final sample predicted and actual values calculated by MLR and RF respectively. The distribution of observed and predicted values of MLR had more residuals than RF. The R^2^ of MLR was 0.58, which was smaller than that of RF (R^2^ = 0.88). The MAE value of MLR (13.25) was higher than that of RF model (4.81). The model interpretation degree of RF was higher than that of MLR, indicating that the deviation error of prediction value of random forest was smaller than multiple linear regression. In addition, there was 1/3 negative value in the urban land prediction value of MLR, which is contrary to the actual significance of urban land spatial change intensity, but there was no negative value in RF. Therefore, the prediction of RF had a larger R^2^ than MLR, the correlation between the actual value and the predicted value was stronger and the model interpretation degree was higher, thus RF was prone to more practicality and it could be used to analyze the factors of urban land expansion.

### Partial dependence for important variables

**Figure 7:**
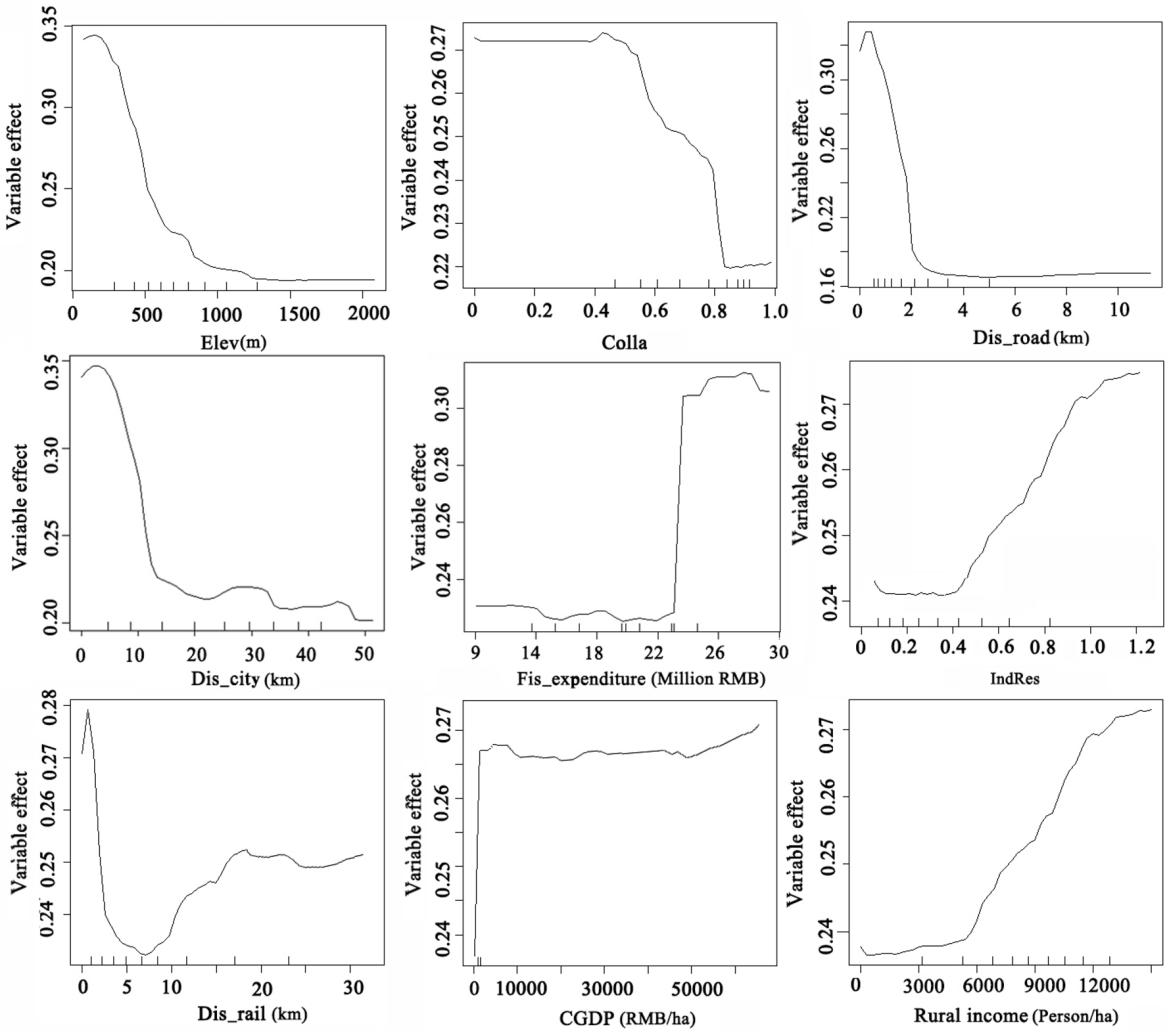
Partial dependence plots of the main influence factors on urban land expansion using RF.

In order to analyze the influence of the regularity of main driving factors on urban land spatial expansion, we used the partial dependence plots to draw the local dependence diagram between urban land expansion and its important influential factors including elevation, risk of goaf collapse area, distance to main roads, distance to city center, distance to railways, local fiscal expenditure, industrial restructring, per capita GDP and per capita rural income (Fig. 7). Urban land area decreased sharply when the elevation was 25 m and the risk of goaf collapse was 0.4, and then stabilized after the elevation was 1300 m and the risk of goaf collapse is 0.8. The distance to roads and distance to city center had a decreasing trend on urban land expansion. When the distance to main roads was 1 km and the distance to city center was 5 km, their impact on urban land was the greatest. When the distance to roads was 3 km and the distance to the city center was 13 km, the impact of those two variables on urban land was minimal and remained unchanged. While the distance to railways showed a trend from decline to rise. Urban land area was mainly distributed in areas with the local fiscal expenditure of 22 million RMB. As the local fiscal expenditure increased, its impact on the expansion of urban land area became greater and reached a peak at 27 million RMB. The industrial restructuring and per capita rural income had a similar trend which was positively correlated and had a stronger effect on urban land expansion. The influence of CGDP was greatest at 11,000 RMB, and then increased after CGDP was 15,000 RMB.

## Discussion

Unlike many studies aimed at the descriptive analysis of urban land expansion, we explored the importance and influence of each variable on urban land expansion using MLR and RF models. The random forests had a better ability (with a high prediction accuracy) in the estimation of urban land expansion change caused by natural and human activities, and 9 variables were identified as important factors in both methods. Among these factors, elevation and the risk of goaf collapse were identified as important geological factors of Mentougou, which is in line with findings of earlier studies^18,49^. Tian et al.^32^ concluded that mining towns had complex topographical conditions and large fluctuations in elevation and goaf collapse area, thus, urban land construction was relatively difficult, which required more technical support and capital investment to expand urban space. However, our study did not find the impact of slope and aspect factors on urban land expansion in both MLR and RF, which is contrary to Cheng^18^. This may be due to most of the urban construction in the study area is located in flat terrain, and the spatial heterogeneity of slope and aspect is inapparent on urban land expansion.

In addition to topographic conditions, the accessibility factors including the distance to city center, to main roads, to railways also significantly influence urban land expansion, which is supported by other studies^20,51^. Our results revealed that the longer distance to main roads and city center, the smaller impact on urban land expansion. Poelmans and Van^52^ believed that the closer to city center, main roads, the more likely a region will be urbanized. However, the partial dependence result of the distance to railways showed a trend of decreasing and then increasing. This may be due to the soil near and traffic lines are loose and weak, which may lead to the expansion of urban area far away from railways^7^.

Our study also identified the importance of land market factors including the per capita GDP and per capita rural income on urban land expansion based on two models. Some studies have noted that the relationship between GDP growth and land expansion is U-shaped, suggesting that land expansion first increases as GDP increases and then decreases beyond a threshold^20^. Regional economy development will increase the investment in urban infrastructure construction, so as to expand domestic demand and speed up the expansion of urban land^6^. According to the result of RF and MLR, per capita GDP and rural income are positively related to urban land expansion in Mentougou, which is consistent with other studies^18,25^.

The financial investment and industrial restructuring in urban construction land, representing government effectiveness, significantly expanded the urban land of Mentougou. Similarly, Galinato et al. ^53^ noted that higher fiscal input is associated with larger urban land scales. In the past decades, the Chinese central government and local governments had invested a large amount of money in the development of urbanization, which directly influenced urban land change in many regions, such as Mentougou. National policies also had a large impact on land management practices and government’s behavior could be seemed as the original impetus for urban land expansion. For example, in 2006, the town’s government started a project that was designed to improve urban land utilization and living environment. Under this project, the government provided reconstruction and compensation to households that were staying in old residential areas and shantytowns in the goaf area, and made great efforts to increase investment in urban infrastructure, urban roads, etc^18^. Additionally, in order to allocate land resources reasonably, local governments also promulgated economic development policies (e.g., city planning) and related plans (e.g., traffic planning)^31^. As of 2010, residential land and traffic land in Mentougou increased by 2034 ha and 1027 ha compared to 2000, respectively. Moreover, the industrial economy of Mentougou was gradually transforming into tourism, ecological agriculture and high-tech industries. However, it should be noted that the increase of average *UE* and average *UI in* 2000–2010 was 4 times that in 1990–2000. This is because most construction land distributed mainly in the plain areas with flat terrain and favorable engineering geological conditions, which combined economic development policies support and financial investment, accelerates population growth and urban expansion^32^. In general, although the increased rate of urban land expansion had slowed down in Mentougou, we couldn't ignore there is still a tendency of excessive expansion of urban land. Therefore, the arduousness of land planning in Mentougou in the future, and more measures, effort and continuous policy support are needed.

In addition, the comparison of MLR and RF revealed the variance explained by RF was about 20% higher than MLR, which indicated that RF was better than MLR in the model fitting. The importance and ranking results of the main driving factors showed that RF method was not a single test of whether there was a significant linear relationship between a certain impact factor and the urban land expansion, it could analyze the relative importance of factors on urban land expansion, and its advantages lied in each variable can be used multiple times, allowing non-linear relationships and interaction to be included in the model^27^. Moreover, the process sampling inspection of RF can ensure the accuracy and stability of significance tests, which is also an important aspect of the method superior to MLR^54^. However, due to the complicated non-linear effect of the driving factors on urban land expansion, the current research is in the deep exploration stage, and statistical models are still the major research method^55^. Our results also indicate that the factors such as distance to the road, the risk of goaf collapse, per capita GDP, and per capita rural income had different orders in both MLR and RF, the reason for the difference was possibly attributable to the differences in statistical principles^27,54^. However, from the existing research, the clarification of these differences is still complicated and uncertain^56,57^, which needs to be further researched.

However, our study still has some limitations, we only selected 16 potential factors as the explanatory variables. In the case of data availability, it is also need to obtain other relevant factors. Furthermore, for the exploration of spatiotemporal variations in urban sprawl, more accurate and efficient estimation approaches, such as nighttime light (NTL) data^58^, can be used in future research to obtain longer high-resolution data time series. Moreover, in the near future, researchers could also focus on exploring the spatiotemporal differences of driving factors in different regions, so as to assess the applicability of the methods for identifying the relative importance of different variables.

## Conclusion

Our research provided a new perspective through machine-learning techniques for ranking the main driving factors of urban land expansion in Mentougou district. Further, we interpreted the land use/cover remote sensing images based on long-term Landsat and GEE platform, which compensates the disadvantage of the low overall accuracy of publicly available land use products, and provides a more effective approach for land mapping. This joint SVM-Machine learning approach is a useful guideline for enriching the existing research framework and predicting the impact on important driving factors of land use/cover change. We found that there was an expanding trend of urban land from 1990 to 2016, and the expansion of urban land area was mainly determined by the combination of natural environment and socio-economic factors. Based on the results here, it needs to design different land schemes according to the importance of explanatory variables, so as to control urban sprawl and minimize the adverse impacts in Mentougou. Further, factors such as elevation, the risk of goaf collapse, government drivers, accessibility, GDP, rural income and population density should be given priority in land use policies.

## Supplementary Information

Supplementary Information.
