# Supplementary material for: Identifying driving factors of urban land expansion using Google Earth Engine and machine-learning approaches in Mentougou District, China
Source: Sci Rep. 2022 Sep 28;12:16248. doi: 10.1038/s41598-022-20478-z (PMC9519550; doi:10.1038/s41598-022-20478-z)
Supplement: Supplementary file 1 — Supplementary Information. [file 41598_2022_20478_MOESM1_ESM.docx]

**Appendix A**

**Table1**. Land use types description, training and verification samples of Landsat TM image

| Types | Landsat  image | Description | Training  samples | Verification  samples |  |
| --- | --- | --- | --- | --- | --- |
| Arable land | **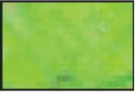** | Displayed in green and grayish green, characteristic as regular geometry and clear boundary | 670 | 447 |  |
| Forestland | 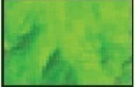 | Displayed in green and dark green, characteristic as fine texture, Uniform color | 2056 | 1371 |  |
| Grassland | 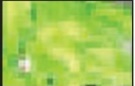 | Displayed in green and light green | 1134 | 756 |  |
| Urban land | 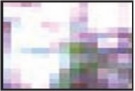 | Displayed in bright white, presented a sporadic distribution | 996 | 664 |  |
|  | 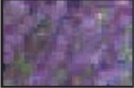 | Displayed in dusty pink, presented a flake distribution |  |  |  |
| Water bodies | 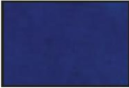 | Displayed in dark blue, presented a curved ribbon | 536 | 357 |  |
| Unused land | 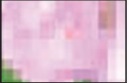 | Displayed in rose hermosa, presented a block distribution | 608 | 405 |  |

^1^ For a sample of Landsat TM image in year 2016

**Table 2**. The area of different land use types in 1990-2016 (ha)

| Type | 1990 | 2000 | 2010 | 2016 |
| --- | --- | --- | --- | --- |
| Arable land | 9404.1 | 7927.29 | 6323.13 | 5983.47 |
| Forestland | 121481.9 | 122050.1 | 120670 | 120072.6 |
| Grassland | 9547.29 | 9320.49 | 8399.43 | 8388.99 |
| Water bodies | 909.99 | 918.54 | 734.76 | 735.48 |
| Urban land | 2900.37 | 4027.56 | 8100.2 | 8997.79 |
| Unused land | 53.91 | 53.55 | 70.83 | 70.74 |
